# ALIX Is Recruited Temporarily into HIV-1 Budding Sites at the End of Gag Assembly

**DOI:** 10.1371/journal.pone.0096950

**Published:** 2014-05-16

**Authors:** Pei-I Ku, Mourad Bendjennat, Jeff Ballew, Michael B. Landesman, Saveez Saffarian

**Affiliations:** 1 Department of Physics and Astronomy, University of Utah, Salt Lake City, Utah, United States of America; 2 Center for Cell and Genome Science, University of Utah, Salt Lake City, Utah, United States of America; 3 Department of Biology, University of Utah, Salt Lake City, Utah, United States of America; University of Alabama at Birmingham, United States of America

## Abstract

Polymerization of Gag on the inner leaflet of the plasma membrane drives the assembly of Human Immunodeficiency Virus 1 (HIV-1). Gag recruits components of the endosomal sorting complexes required for transport (ESCRT) to facilitate membrane fission and virion release. ESCRT assembly is initiated by recruitment of ALIX and TSG101/ESCRT-I, which bind directly to the viral Gag protein and then recruit the downstream ESCRT-III and VPS4 factors to complete the budding process. In contrast to previous models, we show that ALIX is recruited transiently at the end of Gag assembly, and that most ALIX molecules are recycled into the cytosol as the virus buds, although a subset remains within the virion. Our experiments imply that ALIX is recruited to the neck of the assembling virion and is mostly recycled after virion release.

## Introduction

As the HIV-1 Gag protein concentration increases in the cytosol, the protein polymerizes on the plasma membrane to create nascent virions. HIV-1 Gag alone can create fully formed vesicles that bud into the extracellular space as virus like particles (VLPs), implying that the major structural protein of the virus contains all of the information necessary for particle assembly and budding [1]. The Gag polyprotein comprises four elements: an N-terminal MA domain that is myristylated and binds PI(4,5)P_2_ molecules on the inner leaflet of the plasma membrane, a central CA region that makes important protein–protein interactions during virion assembly, an NC domain that captures the viral RNA genome during assembly and a C-terminal p6 region that is essential for budding [2], [3], [4], [5]. Two short peptide motifs in the p6 region, termed late assembly domains, are required for efficient budding of infectious virions [6], [7], [8]. These motifs function by interacting directly with early acting components of the endosomal sorting complexes required for transport (ESCRT) pathway. Specifically, the p6^Gag^ PTAP motif interacts with the TSG101 subunit of the ESCRT-I complex [9], [10], [11], [12] and the YPX_n_L motif interacts with ALIX [13], [14], [15], [16], [17]. ALIX also interacts with upstream NC^Gag^ elements [18], [19]. In most cell types, the PTAP element appears to be the dominant late assembly domain [20], [21]. However, mutations that remove the PTAP element can be substantially rescued by ALIX overexpression, implying that ALIX and ESCRT-I can function redundantly, at least in some contexts [22], [23]. Furthermore, some related retroviruses like the Equine Infectious Anemia Virus (EIAV) lack TSG101/ESCRT-I binding sites and appear to bud exclusively through ALIX, implying that ALIX alone can initiate assembly of the ESCRT machinery [13], [14], [15], [16].

ALIX comprises three distinct elements, an N-terminal, crescent-shaped Bro1 domain, a central V-shaped V domain, and a C-terminal proline-rich region (PRR) that is thought to fold back and auto inhibit ligand interactions of the upstream domains [22], [24], [25]. The HIV-1 p6^Gag^ YPX_n_L late assembly domain binds in a pocket on the inner face of the second arm of the V domain [22], [25] and series of positively charged residues on the Bro1 domain interact with NC^Gag^ [26]. In addition to these Gag interactions, a PSAP motif within the ALIX PRR can bind TSG101/ESCRT-I [14], [15], [16], and the second arm of the V domain can bind Lys-63 polyubiquitin chains [27], [28]. The PSAP motif within ALIX has not been shown to contribute to ALIX recruitment to sites of virus budding, but ubiquitin binding contributes to ALIX recruitment and/or bud functions [22], [27], [29]. Current models hold that ALIX is activated through release of PRR auto inhibition and V domain opening. These conformational changes may increase membrane binding affinity [24], [30] and/or induce protein dimerization [31] which promote ALIX binding to ESCRT-III subunits of the CHMP4 family through interactions at its Bro1 domain [22], [31], [32], [33], [34].

Human cells express twelve ESCRT-III proteins that are divided into eight different families (CHMP1-7 and IST1) [35]. Functional and imaging analyses have demonstrated central roles in HIV-1 budding for two of the ESCRT-III families; CHMP4 (three members, CHMP4A-C) and CHMP2 (two members, CHMP2A, B). Two additional ESCRT-III families appear to perform accessory roles; CHMP1 (two members, CHMP1A, B) and CHMP3 [36], [37]. Although mechanistic details are lacking, the ESCRT-III subunits are thought to polymerize into filaments that constrict the bud neck and promote membrane fission [35], [36], [37], [38], [39], [40], [41]. The ESCRT-III filaments also recruit essential AAA ATPase, VPS4, which is thought to remodel and/or disassemble the filaments [42], [43], [44]. VPS4 is essential for HIV budding and is recruited at the final stages of virion assembly [35], [45], [46], [47].

In recent years, imaging techniques such as total internal reflection fluorescence microscopy (TIR-FM) have been used to visualize viral assembly and budding from living cells. TIR-FM provides excellent spatial and temporal resolution and is ideally suited for visualizing HIV-1 assembly at the plasma membrane because the excitation field at the coverslip-cell interface decays exponentially, and therefore only excites fluorophores that are within about 100 nm of the plasma membrane [48], [49], [50]. This property eliminates confounding background fluorescence from molecules that are deeper within the cell.

TIR-FM has been used to define many dynamic features of retroviral assembly including virion assembly [47], [51], [52], viral genome capture [53], and ESCRT protein recruitment [45], [46]. Elegant TIR-FM studies of HIV-1 and EIAV assembly have shown that the ESCRT-III and VPS4 proteins arrive late, after Gag assembly is complete and immediately preceding the budding event. As the VLP buds, the ESCRT-III and VPS4 proteins appear to be released back into the cytoplasm rather than remaining within the virion [45], [46], [47]. A recent super resolution imaging study has challenged this model, however, arguing instead that the bulk of the recruited CHMP4B remains within the released virion [54]. This discrepancy has important mechanistic implications for HIV-1 budding because CHMP4B retention implies that the ESCRT-III assembly constricts the bud neck from the virion-proximal side, whereas release of ESCRT-III proteins back into the cytoplasm implies that the ESCRT-III filaments constrict the bud neck from the cell-proximal side.

In contrast to the late-acting ESCRT factors, the dynamic recruitment of the early-acting ESCRT proteins remains less well characterized because previous experiments have lacked the sensitivity required to visualize ALIX or ESCRT-I recruitment to sites of HIV-1 budding. It has, however, been possible to visualize ALIX recruitment to sites of EIAV Gag assembly [45], presumably because ALIX binds more efficiently to the YPX_n_L motif of EIAV p9^Gag^ than to the one of HIV-1 p6^Gag^ [55]. In the EIAV Gag studies, ALIX recruitment was seen to initiate early and accumulate steadily in parallel with Gag accumulation [45]. Unlike ESCRT-III and VPS4, most of the ALIX molecules remained associated with the budded EIAV virions. Thus, the dynamic recruitment profile for the early-acting ALIX factor appeared to be very different from that of the late-acting ESCRT-III and VPS4 factors.

The importance of HIV-1 as both a medical problem and as the leading model system for studying virus budding prompted us to attempt to visualize ALIX recruitment to sites of HIV-1 budding. Using a fluorescently-labeled ALIX protein that is fully functional in HIV-1 budding, we were able to visualize ALIX recruitment to sites of HIV-1 Gag VLP assembly. The recruitment profiles revealed that ALIX is recruited late in the assembly process and that most, but not all, of the ALIX molecules are released back into the cytoplasm upon VLP release.

## Materials and Methods

### Infectivity Assay

Virions were produced in 293T cells (4×10^5^ cells/well in 6-well plates) following calcium-phosphate (Clontech) co-transfection of the following plasmids: 1.35 µg of an HIV RΔ8.2 construct (either wild-type or incorporating a _7_LIRL_10_ instead of _7_PTAP_10_ in the p6^Gag^ (ΔPTAP) [20]), 1.35 µg of pLOX-GFP [56], 0.4 µg of pCMV-VSV-G and 1 µg of the negative control empty vector (pCI-FLAG-EV) or ALIX expression vector. The medium (2 ml/plate) was replaced 8 hr after transfection, and the supernatant was harvested 16 hr later and syringe-filtered through 0.45 µm membranes. Viral titers were measured using flow cytometry/fluorescence-activated cell sorting (FACS) to detect green fluorescent protein (eGFP) expression from the packaged pLOX-GFP vector in transduced HeLa cells. For western blotting, cells were harvested with cold PBS and lysed in 200 µL of RIPA (50 mMTris, 150 mM NaCl, 0.1% SDS, 0.5% sodium deoxycholate, 1% NP40 and complete protease inhibitor cocktail (Sigma)). Virions were collected by pelleting 1 mL of medium through a 250 µL 20% sucrose cushion. The supernatant was removed and the virion pellet was dissolved in 60 µL of SDS-PAGE loading buffer. Western blots were performed using anti-CA, 1∶3000 (NIH AIDS Research & Reference Reagent Program, Catalog #3537) and anti-ALIX 1∶5000 [22].

### Cell Culture and Transfection

293T and HeLa cells were maintained in Dulbecco’s modified Eagle’s medium supplemented with fetal calf serum (10%), sodium pyruvate (1 mM) and L-glutamine (2 mM). HeLa cell lines stably expressing eGFP-tagged ALIX were generated by transfection with Lipofectamine 2000 (Invitrogen), followed by Geneticin (G418, Invitrogen) selection (0.5 mg/mL) and flow cytometry sorting. Single-cell clones expressing the proper levels of the fusion protein were selected for experiments. For TIR-FM experiments, HeLa cells (100×10^3^ cells/well in 25 mm plates) were transfected with untagged Gag and Gag-mCherry in a 5∶1 ratio using Lipofectamine 2000.

#### Microscope description

Live images were acquired using iMIC Digital Microscope made by TILL photonics controlled by TILL’s Live Acquisition imaging software as previously described [47]. Two wavelengths of laser, 488 nm diode laser (Toptic photonics, iBeam smart 488S) and 561 nm diode-pumped solid state (DPSS) laser (Cobolt Jive, 561 nm Jive High Power), were used to excite eGFP and mCherry, respectively. Laser beams passed through an AOTF (acousto-optical tunable filter) and focused into a fiber which delivers the light to TILL Yanus digital scan head and then Polytrope II optical mode switch (Diagram shown in supplementary materials). Yanus consists of two galvo-mirrors and one spherical mirror to control the laser beam position. The Polytrope rapidly switches illumination beam path between Epi (widefield), FRAP and TIRF microscopy modes. It also holds the quadrant photodiode used for TIRF penetration depth calibration, which was set to 150 nm for the experiments in this manuscript. In the TIRF mode Yanus is used to control the position of the focused beam in the objective’s back focal plane and can be adjusted within 0.2 ms. We positioned the focused beam at the edge of the back focal plane of the objective (N = 1.46, 100X, Zeiss) to reach beyond the critical angle and achieve TIRF. TIRF critical angle was verified by scanning the laser beam across the back aperture and measuring the reflection of the laser from the Glass sample interface back into the objective and onto the quadrant photodiode [47]. The penetration depth of the beam is calculated based on the incident angle of the beam which is in turn measured by the position of the beam on the quadrant photodiode. Once the penetration depths for the experiments are set at the beginning of acquisition, a feedback loop keeps the focus of the objective on the sample by constantly monitoring the position of the back reflected beam with respect to the original beam. We also rotated the TIRF illumination on the objective back focal plane 1 turn/exposure (TIRF360) to maximize homogeneity of the TIRF images.

## Results

### ALIX Linked to eGFP at its C Terminus with a 30 Amino Acid Helical Linker Supports Production of HIV-1 with Equal Infectivity as Wild Type ALIX

**Figure 1:**
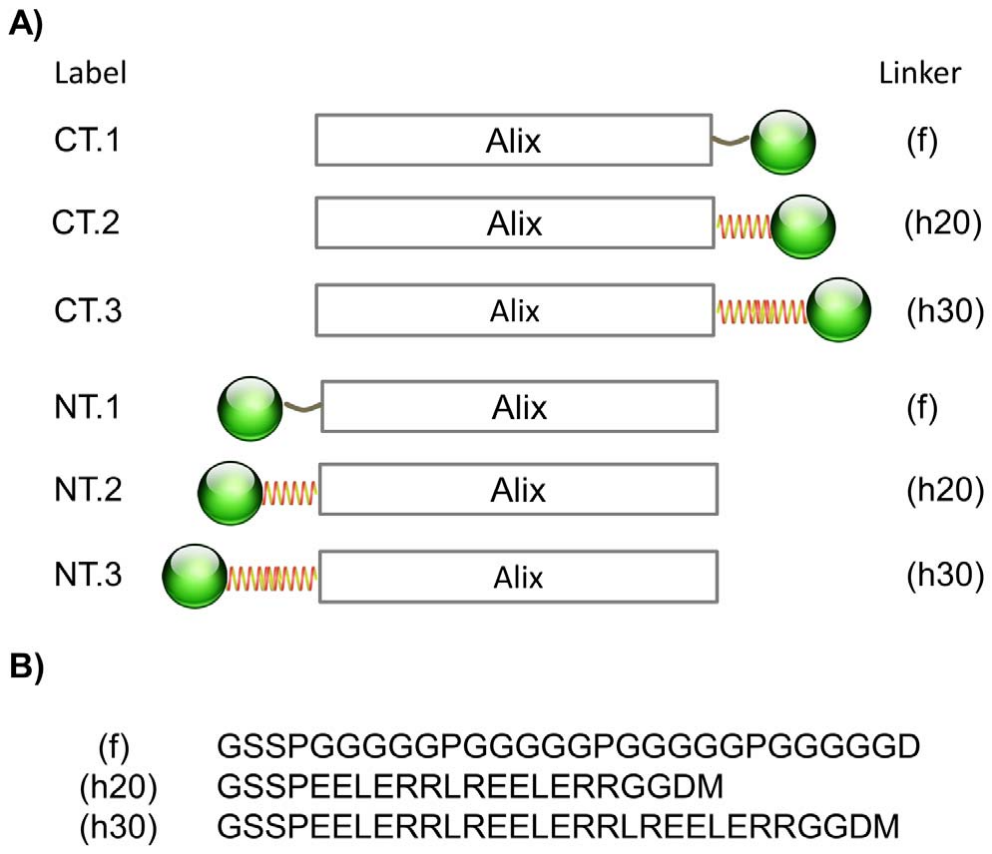
Diagram representation of ALIX eGFP fusion protein library. ALIX was fused to eGFP using three designed linkers (f, h20 and h30). (A) Shows the designation of the fusion proteins with the orientation of the linker fusions. (B) The amino acid sequence of the three linkers.

Our goal was to use fluorescently labeled proteins to image how ALIX is normally recruited to sites of HIV-1 Gag assembly, and we therefore began by testing different ALIX-eGFP constructs for the ability to function normally in virus budding. Six DNA constructs were created that encoded the joining of ALIX to eGFP with protein linkages of materially different character. The rationale behind constructing linkers was to create some distance between eGFP and ALIX. However, since it is not clear how ALIX fits into the functioning ESCRT machinery, a library of linkers was created so that various lengths and orientations may be tested. DNA sequences were generated using two helical linkers of similar composition, but of varying length (20 and 30 amino acids), and one flexible linker of 28 amino acids in length [57]. As shown in Figure 1, all three linkers were used to generate ALIX fusions with eGFP in both gene orders, *i.e.* eGFP before ALIX and ALIX before eGFP, for six total linkage combinations.

**Figure 2:**
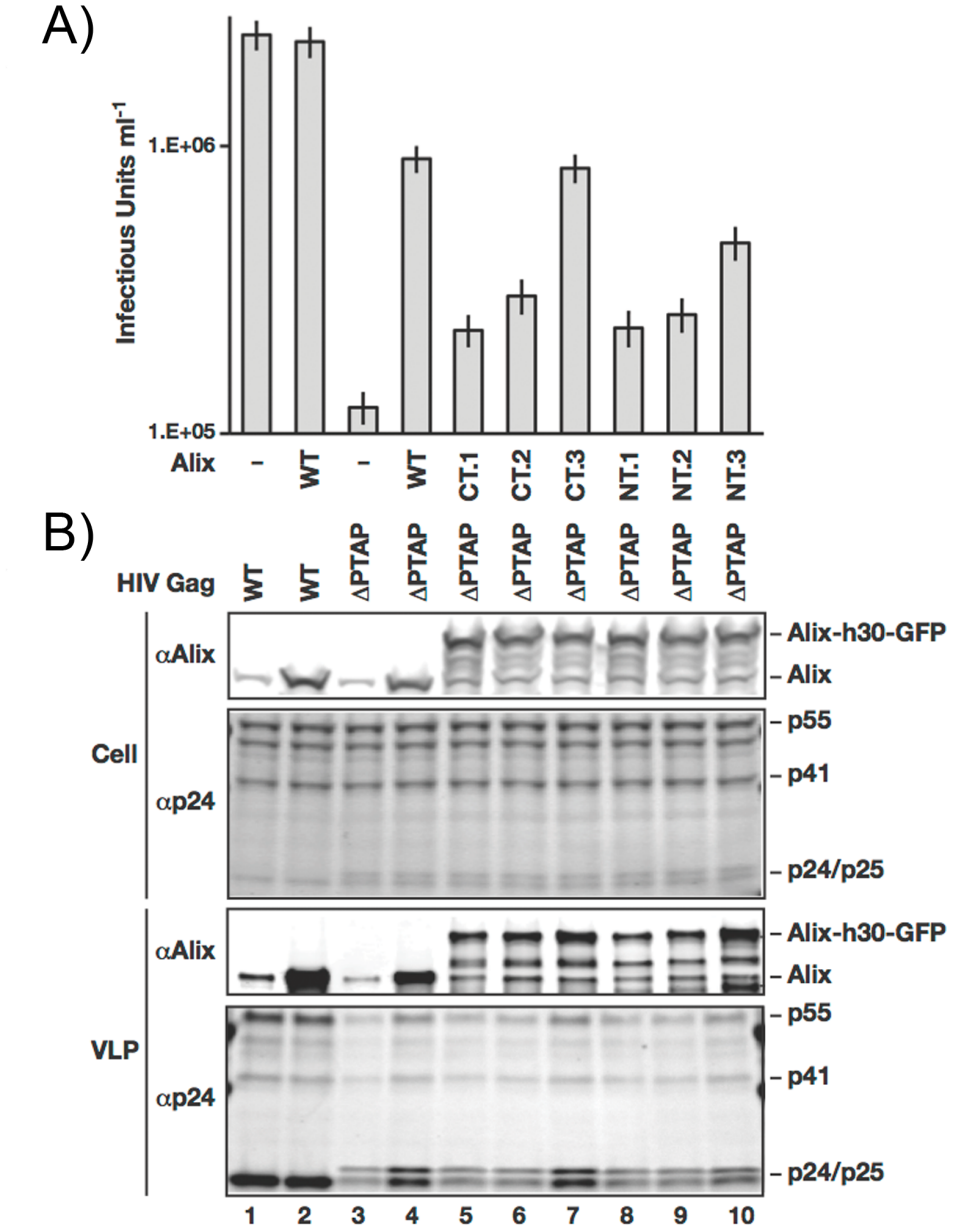
Characterization of the ALIX fusion proteins with eGFP. A) Cells expressing ALIX fusions (lanes 5–10), wt ALIX (lanes 2 and 4) or control (lanes 1 and 3) where infected with WT (Lanes 1 and 2) as well as ΔPTAP HIV-1 RΔ8.2 (Lanes 3–10). Assembled virions were harvested 16 hrs post infection and viral titers were measured using FACS to detect eGFP expression from the packaged pLOX-GFP vector in transduced HeLa cells. B) Western blot analysis of cells and collected virions from the same experiment presented in A.

Infectivity of HIV-1 virions is significantly affected by a ΔPTAP mutation (motif _7_LIRL_10_ instead of _7_PTAP_10_ [20]). This inefficiency, which is due to inability to recruit TSG101, can be rescued by over expressing ALIX [22], [23]. We used this system to test the proper assembly of HIV-1 ΔPTAP in the presence of the six ALIX fusion constructs. Figure 2A shows the infectivity of HIV-1 ΔPTAP virions harvested from 293T cells under over expression of various ALIX fusion proteins; the constructs are over expressed to similar levels as shown in Figure 2B. The ALIX-h30-eGFP (CT.3 #7) as shown in the figure supports assembly of HIV-1 ΔPTAP virions with equivalent infectivity to wild type ALIX. Western blots on harvested virions showed incorporation of ALIX-h30-eGFPas shown in Figure 2B.

**Figure 3:**
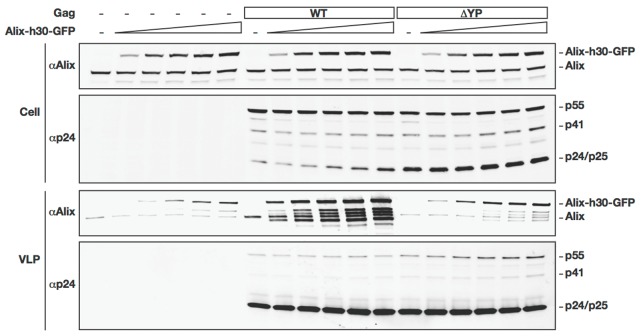
Incorporation of ALIX-h30-eGFP is dependent on the Gag late domains. 293T cells were transfected with either RΔ8.2 variants (WT or ΔYP) alone or with increasing amounts of the ALIX-h30-GFP expressing vector. 24 h post-transfection, cell lysates and VLPs were analyzed by western blot using the indicated antibodies.

We further tested the specificity of incorporation of ALIX-h30-eGFP into VLPs by transfecting ALIX-h30-eGFP and HIV-1 ΔYP into 293T cells. As shown in Figure 3, the incorporation of ALIX into VLPs was dependent on its expression level and is substantially diminished in VLPs produced by HIV-1 ΔYP mutant (YP to SR mutation [14]).

**Figure 4:**
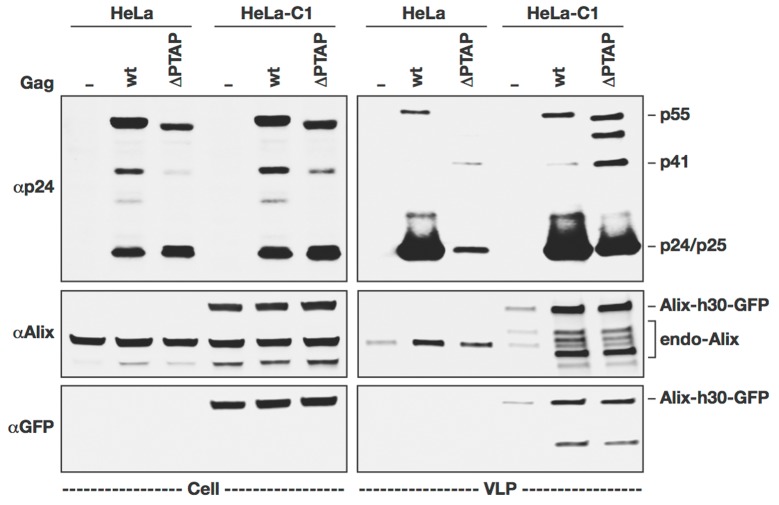
VLP formation in HeLa-C1 cells stably expressing ALIX-h30-eGFP. HeLa and HeLa-C1 cells were transfected with ΔR8.2 variants (WT or ΔPTAP). 24 h post-transfection, cell lysates and VLPs were analyzed by western blot using the indicated antibodies.

To image the incorporation of ALIX into forming VLPs, we proceeded with creating a stable cell line expressing ALIX-h30-eGFP. The ALIX-h30-eGFP was transfected into Hela cells and used to create a cell line. This cell line was then cloned and cells from a single clone were grown for experiments. In this clone, ALIX-h30-eGFP is expressed stably at a similar level of expression when compared to wild type ALIX as shown in Figure 4. A minimal amount of ALIX as well as ALIX-h30-eGFP is released from these cells in the absence of Gag due most likely to cellular basal microvesicle formation. This clone, named HeLa-C1, was further used in the imaging experiments.

### ALIX Incorporates Transiently at the End of Gag Assembly during the Formation of HIV-1 VLPs

**Figure 5:**
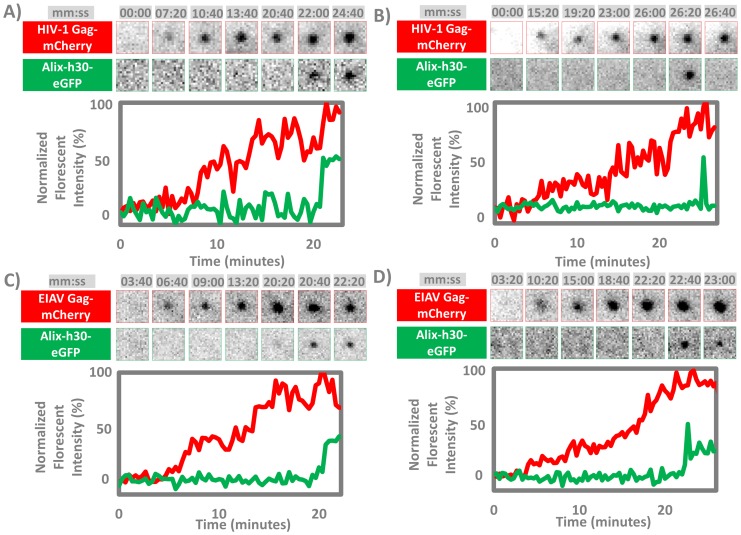
Incorporation of ALIX-h30-eGFP into forming HIV-1 and EIAV VLPs. HeLa-C1 cells stably expressing ALIX-h30-eGFP were transfected with either HIV-1 Gag and Gag-mCherry plasmids (A and B) or EIAV Gag and Gag-mCherry plasmids (C and D). In all, ALIX arrives at or near the completion of Gag assembly. In HIV-1 case, we observed 44 VLP assembly events incorporating ALIX; 34 out of 44 events were similar to B in which no residual ALIX was detected after the initial transient recruitment, and 10 out of 44 events were similar to A in which some ALIX was left in the VLP. In EIAV case, we observed 33 EIAV VLP assembly events; retention of the ALIX is significantly higher in EIAV with 18 events that retained almost all of the ALIX as shown in D.

**Figure 6:**
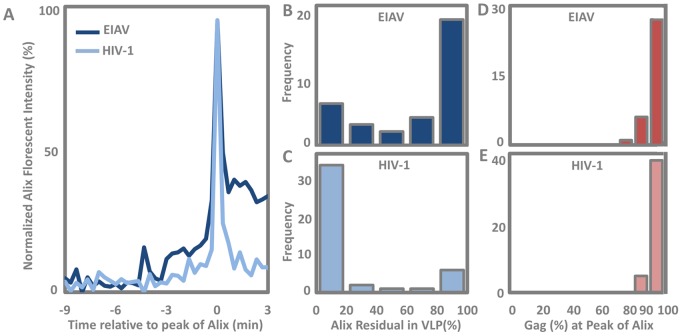
Characterization of ALIX recruitment. A) Temporal averaging of ALIX fluorescence intensity, with all profiles lined up so the peak recruitment is positioned at time zero. This averaging shows early ALIX recruitment up to 5 minutes before appearing as a spike. The retained ALIX is also visible in HIV-1. B) Distribution of ALIX retention in EIAV VLPs. C) Distribution of ALIX retention in HIV-1 VLPs. (D and E) show percentile of total accumulated Gag within the VLP at the time of peak ALIX recruitment for EIAV and HIV respectively.

To observe incorporation of ALIX into forming HIV VLPs, the HeLa-C1 Cells were transfected with a mixture of Gag and Gag-mCherry plasmids as explained in methods. HIV-1 Gag VLPs were observed forming between 3–5 hrs post transfection. Cells were imaged for 90 minutes with a frame rate of 20 sec/frame. TIRF images in both 488 and 561 channels corresponding to ALIX-eGFP and Gag-mCherry were acquired independently with 100 mSec in between the two acquisitions. As shown in Figure 5 (panels A and B) and movie S1, HIV-1 VLPs nucleated on the plasma membrane and the fluorescence intensity grew over time indicating Gag polymerization and incorporation within the VLP. When Gag polymerization was complete the fluorescence intensity detected from the VLP reached a plateau indicating completion of assembly [47]. We analyzed the incorporation of Gag and ALIX in 44 VLPs from 5 cells, in all events ALIX-h30-eGFP was recruited into the VLP near the final plateau of Gag assembly. To better determine the relationship between the incorporation of ALIX and Gag polymerization kinetics, we used the shell filling model developed previously to fit the Gag polymerization profiles to determine the fluorescence intensity at the completion of assembly [47]. As shown in Figure 6E, based on this analysis, in 40 out of 44 observed VLPs, ALIX-h30-eGFP was recruited when 100% of the Gag was assembled, in 4 out of 44 VLPs, ALIX-h30-eGFP was observed when 90% of the Gag was assembled and lower percentiles were not observed. As shown in Figure 5 (panels A and B), two distinct behaviors of ALIX were observed. In 34 of the 44 observed VLPs less than 20% of recruited ALIX was retained within the VLP after fission, this leftover ALIX was not reliably detectable given the background fluorescence. 10 of the 44 observed VLPs retained a detectable fraction of the ALIX-h30-eGFP (Figure 6C). To further investigate the timing of recruitment of ALIX, we transfected Hela-C1 cells with VPS4-h37-mCherry [47] and HIV-1 Gag expressing plasmids. In these cells the recruitment of ALIX is observed overlapping with VPS4 as shown in Figure S1. The temporal resolution of 15 seconds used in these experiments however, is not sufficient to resolve the relative timing of recruitment of ALIX with respect to VPS4.

### ALIX Incorporates Transiently at the End of Gag Assembly during the Formation of EIAV VLPs

EIAV assembly is driven by EIAV Gag which has a very similar domain distribution to HIV-1. EIAV however has a p9 domain that lacks the PTAP TSG101 binding motif, and instead binds strongly to ALIX through its YPX_n_L motif. The affinity of EIAV p9^Gag^ to ALIX is higher than that of HIV-1 p6^Gag^ [55]. To measure the recruitment of ALIX into EIAV Gag VLPs, we imaged the assembly of EIAV VLPs in the Hela-C1 cell line expressing ALIX-h30-eGFP. As shown in Figure 5, panels C and D and movie S2, polymerization of EIAV Gag reaches a plateau similar to HIV-1 Gag polymerization, at which time ALIX-h30-eGFP is observed recruiting into the budding VLP. We analyzed the incorporation of EIAV Gag and ALIX in 33 EIAV VLPs in 5 cells, and in all events ALIX-h30-eGFP was recruited into the VLPs near the final plateau of Gag assembly (Figure 6D). In 6 of the 33 observed VLPs less than 20% of recruited ALIX was retained within the VLP; this leftover ALIX was not reliably detectable above the background. 27 of the 33 observed VLPs, however, retained a detectable amount of ALIX-h30-eGFP, with 18 retaining more than 80% of recruited ALIX, as shown in Figure 6B. The enhanced retention of ALIX within EIAV Gag VLPs is consistent with the higher affinity of EIAV p9^Gag^ compared to HIV-1 p6^Gag^.

### Comparison between ALIX-h30-eGFP Recruitment to EIAV and HIV-1 Gag during VLP Assembly

While it is challenging to observe ALIX recruitment before the peak of recruitment in individual profiles, we aligned all the ALIX fluorescence intensity profiles based on the position of the recruitment peak of ALIX to show the averaged intensity from all profiles (Figure 6A). Based on this averaging we can observe a slight linear recruitment of ALIX as early as 5 minutes prior recruitment peak.

ALIX recruitment to both HIV-1 and EIAV Gag is transient and at the end of Gag polymerization and the EIAV and HIV-1 profiles are only different in terms of the retention level of ALIX within the VLPs (Figure 6). We defined the retention factor as the ratio of maximum intensity reached by ALIX at the peak of recruitment to the intensity of ALIX remaining in the VLP after the initial transient recruitment. Figure 6 demonstrates that while the retention factor for HIV-1 is 10%, the EIAV Gag has a much higher average retention factor of 40%, which is consistent with the stronger affinity of the EIAV p9 region for ALIX compared to that of HIV-1.

## Discussion

Our data shows that ALIX is recruited transiently into forming HIV-1 as well as EIAV VLPs after polymerization of Gag is complete. ALIX interacts directly with Gag through the p6^Gag^ [22], [25], NC^Gag^ [18], [19], [26] as well as indirectly through ubiquitin binding [27], [28], [29] and TSG101 [14], [15], [16]. Since Gag is abundant within the VLP, the sudden recruitment of ALIX into formed VLPs suggests that some or all of interactions between Alix and Gag are modulated during assembly in vivo.

The observed ALIX recruitment at the end of Gag assembly supports a model of recruitment where ALIX is recruited to the neck of the formed VLP. It was shown by imaging ESCRT recruitment onto giant unilamellar vesicles (GUV) that during invagination of membrane, elements of ESCRT I and II proteins are localized to the periphery of the invagination and remain on the cytosolic side of the membrane even after secession of the formed vesicle [58]. These experiments are conducted in the context of the MVB pathway and the function of ESCRT machinery in MVBs is dominated by ubiquitin binding. Early studies identified a link between ubiquitin and retrovirus release [59], [60], [61] and recent reports are unraveling more about the role of ubiquitin in HIV-1 budding. Indeed, ubiquitin ligases were clearly shown to influence the budding of HIV-1 [62], [63], [64] and ubiquitin conjugation to Gag appears to be essential for ESCRT mediated HIV-1 budding [29], [65]. Interestingly, ALIX binds specifically to ubiquitin through its V domain [27], [28], [66]. It is therefore possible that ubiquitin linked interactions might assist/control the specific recruitment of ALIX onto the neck of the fully assembled Gag lattice during virus budding. While this hypothesis is attractive and it is known that ALIX binds ubiquitin, the ubiquitinated protein(s) that bind ALIX remain to be identified. The effect of ubiquitin binding on ALIX also remains unclear. ALIX can be activated through opening of its V domain and potential dimerization which results in higher affinity for the membrane and CHMP4 [24], [30], [31], it is also possible that activation of ALIX might play a role in its recruitment to the neck of the forming VLP.

An ALIX fusion with GFP at the Bro1 domain was shown to recruit from the beginning of the EIAV Gag assembly [45]. The Bro1 domain fusion to GFP however exhibits defects in infectivity assays [15]. When average recruitment profiles are analyzed from the experiments presented here as shown in Figure 6, our data also supports some earlier recruitment of ALIX during the VLP formation, however this amount is minimal compared to the major recruitment at the end of assembly.

During HIV and EIAV budding, our results show that a portion of ALIX is retained within released virions, with a higher retention rate in EIAV compared to HIV-1, consistently with the higher affinity of EIAV p9^Gag^ to ALIX when compared to HIV-1 p6^Gag^ [55]. The removal of a significant portion of ALIX after initial incorporation into the formed VLP is still puzzling. Initially, we speculated that the loss of ALIX signal is due to self-quenching of eGFP based on the close proximity of packaged eGFPs trapped within the VLP after fission of the membrane [67], [68]. Although we still cannot completely rule out some self-quenching effects, given that EIAV has very similar VLP size to HIV-1 and the ALIX signal at the end of EIAV assembly is mostly retained, the self-quenching of eGFP cannot convincingly explain the loss of eGFP signal specially in the HIV-1 case. Therefore it is reasonable to assume that the loss of eGFP signal has to do with dissociation of ALIX from the VLP during and/or after fission of the membrane.

ESCRT III proteins have been shown to polymerize into spiral structures on the plasma membrane [37], [38], [39], it is therefore possible that ALIX would dissociate from the Gag lattice due to the forces applied during either polymerization of CHMP4, CHMP2 or recruitment of VPS4.

## Supporting Information

Figure S1 **Recruitment of VPS4-h37-mCherry along with ALIX-h30-eGFP in Hela-C1 cells.** Cells were transfected with, Gag and VPS4-h37-mCherry plasmids and imaged 5 hrs post transfection. The time resolution of these experiments is 15 seconds and therefore insufficient for separating the recruitment of ALIX from VPS4. In the graph, A shows the recruitment dynamics of one spot, B shows the average recruitment profiles for 22 spots, all of them had both ALIX and VPS4 co recruitment.
(DOCX)

Movie S1 **Recruitment of ALIX into the forming HIV VLPs.** Movie shows ALIX-h30-eGFP (Green) recruitment into two forming Gag assembly sites (Red) as a spike at the end of assembly.
(MOV)

Movie S2 **Recruitment of ALIX into the forming EIAV VLPs.** Movie shows ALIX-h30-eGFP (Green) recruitment into two forming EIAV assembly sites (Red) as a spike with some residual intensity at the end of assembly.
(MOV)
