# Supplementary material for: ALIX Is Recruited Temporarily into HIV-1 Budding Sites at the End of Gag Assembly
Source: PLoS One. 2014 May 16;9(5):e96950. doi: 10.1371/journal.pone.0096950 (PMC4023924; doi:10.1371/journal.pone.0096950)
Supplement: Figure S1 — Recruitment of VPS4-h37-mCherry along with ALIX-h30-eGFP in Hela-C1 cells. Cells were transfected with, Gag and VPS4-h37-mCherry plasmids and imaged 5 hrs post transfection. The time resolution of these experiments is 15 seconds and therefore insufficient for separating the recruitment of ALIX from VPS4. In the graph, A shows the recruitment dynamics of one spot, B shows the average recruitment profiles for 22 spots, all of them had both ALIX and VPS4 co recruitment. (DOCX) [file pone.0096950.s001.docx]

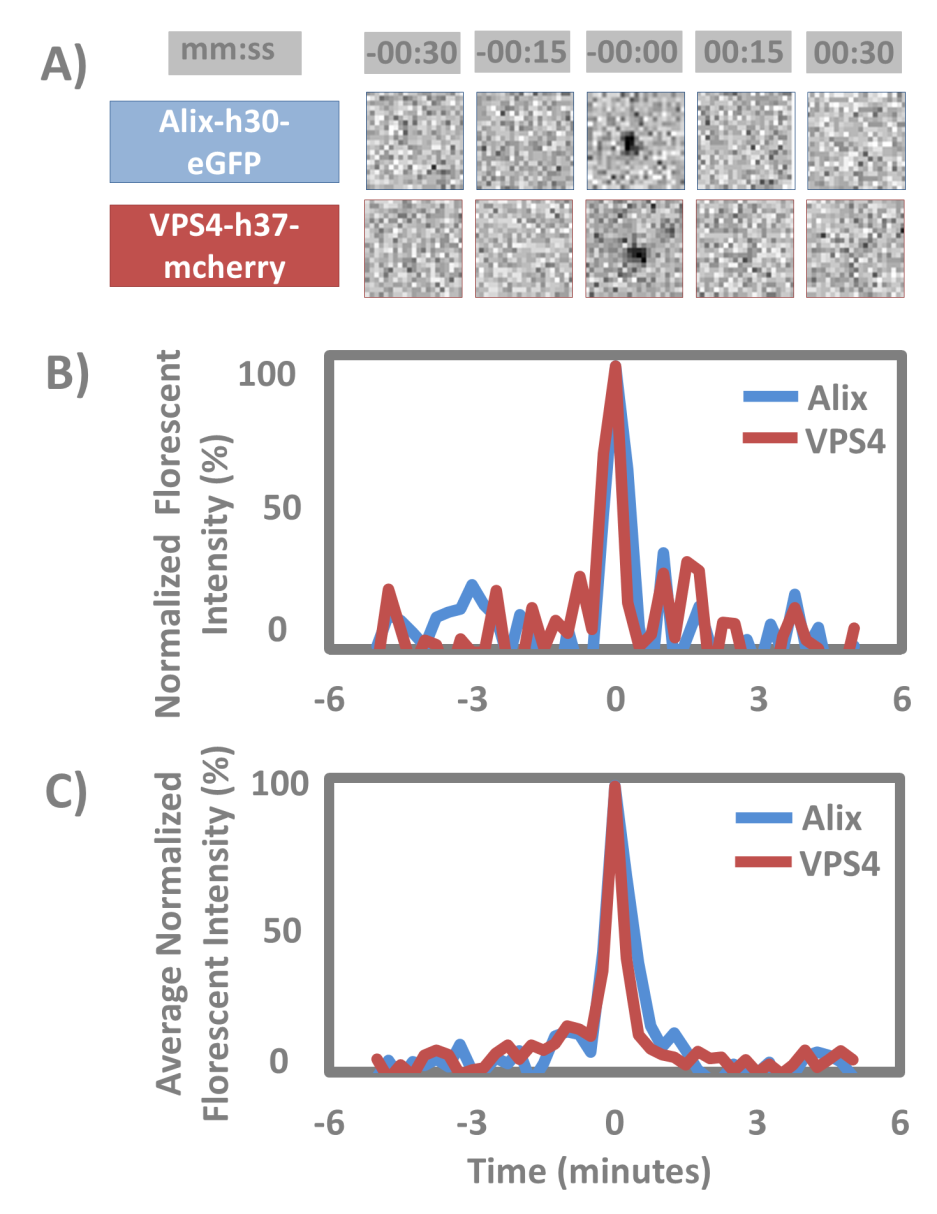


Figure S1: Recruitment of VPS4-h37-mCherry along with ALIX-h30-eGFP in Hela-C1 cells. Cells were transfected with, Gag and VPS4-h37-mCherry plasmids and imaged 5hrs post transfection. The time resolution of these experiments is 15 seconds and therefore insufficient for separating the recruitment of ALIX from VPS4. In the graph, A shows the recruitment dynamics of one spot, B shows the average recruitment profiles for 22 spots, all of them had both ALIX and VPS4 co recruitment.
